# Dissemination of Plastic Surgery Research: An Analysis of *PRS* and *PRS-GO*

**DOI:** 10.1097/GOX.0000000000003808

**Published:** 2022-03-11

**Authors:** Jacob S. Nasser, Jessica I. Billig, Sakura Horiuchi, Kevin C. Chung

**Affiliations:** From * The George Washington School of Medicine and Health Sciences, Washington, D.C.; † Section of Plastic Surgery, Department of Surgery, Michigan Medicine, Ann Arbor, Mich.; ‡ Section of Plastic Surgery, University of Michigan Medical School, Ann Arbor, Mich.

## Abstract

*Plastic and Reconstructive Surgery (PRS*) recently developed an open access counterpart, *PRS Global Open (PRS-GO*), to increase dissemination of research in an efficient and widespread manner. We aimed to (1) examine the differences in the dissemination of research published in *PRS* and *PRS-GO*, and (2) identify differences in the authorship between the journals.

**Methods:**

We extracted data on Altmetric Attention Scores, article mentions, citations, and author characteristics using the Altmetric Explorer Database from January 1, 2018, to January 1, 2020. We stratified research outputs into traditional dissemination and social media dissemination. Additionally, multivariable linear regression models were used to examine differences in dissemination between the journals.

**Results:**

A total of 1798 articles were included in the analysis (*PRS* = 1031, *PRS-GO* = 767). The average Altmetric Attention Score was higher for *PRS* compared with *PRS-GO* (*PRS* = 15.2, *PRS-GO* = 8.1). Articles in *PRS* had a greater Altmetric Attention Score (β-coefficient: 7.50, *P* < 0.001), higher measures of traditional dissemination (β-coefficient: 3.11, *P* < 0.001), and higher measures of social media dissemination than articles in *PRS-GO* (β-coefficient: 4.38, *P* = 0.73).

**Conclusions:**

Despite being an open access journal, *PRS-GO* had significantly fewer measures of social media and traditional dissemination compared with *PRS*. Given that numerous factors may influence the dissemination of scientific literature, it is imperative that publications identify specific ways to provide a fair advantage for both researchers and readers. Additional initiatives to engage readership for open access may include creative campaigns targeting an appropriate audience.

**Takeaways**

**Question:** What are the differences in dissemination between *PRS* and *PRS-GO*? Can a traditional journal use an open access strategy to facilitate transfer of scientific information?

**Findings:** Dissemination was greater for articles in *PRS* compared with *PRS-GO* for period examined, but some measures of dissemination were comparable for more recent *PRS* and *PRS-GO* publications.

**Meaning:** Editorial staff should identify strategies to promote research from authors with lower resources and strategies to increase dissemination for readers consuming the research.

## INTRODUCTION

Recently, there has been a rise in open access (OA) journals. OA journals promise an increase in readership by removing the paywall and providing free access with unrestricted downloading and sharing rights to the public.^1^ On the other hand, traditional, non-OA medical journals are typically associated with fee-based subscription and copyright agreements, thus potentially limiting public access.^2^ Evidence suggests that OA journals are providing content with a similar scientific impact compared with traditional journals.^3^ Nonetheless, the actual dissemination of research in traditional journals compared with their OA counterparts is largely unknown. Journal- and specialty-specific analyses can provide a more comprehensive understanding on the effectiveness of dissemination of research using OA publications in a particular scientific field.

Researchers have previously examined the dissemination of research in various plastic surgery publications using Altmetric Attention Scores (AAS).^4,5^ For instance, Boyd et al compared Altmetric scores with traditional bibliometrics for the 10 most cited articles in the top plastic surgery journals and found the two variables were not strongly correlated.^4^ Additionally, Asaad et al examined AAS and citation counts of the top six plastic surgery journals in 2016, and found that *Plastic and Reconstructive Surgery (PRS*) was the journal with the second highest mean AAS after the Aesthetic Surgery Journal.^5^ Nonetheless, there is a lack of research examining difference in the dissemination of a traditional plastic surgery journal with an OA counterpart.

*PRS* is the highest-impact plastic surgery journal in the world.^6^ In 2013, *PRS Global Open* (*PRS-GO*) was established by removing the paywall and providing unlimited access to plastic surgical publications. Numerous promotional strategies have been implemented to create transparency about the OA model of *PRS-GO* and educate plastic surgeons on the role that the publication model can have on the dissemination of plastic surgery research worldwide.^7,8^ Nonetheless, to date, there is no analysis on the impact of the OA model in dissemination of research within plastic surgery. Although OA publications provide medical professionals and researchers access to medical literature free of charge, critics of OA journals argue that author fees may introduce an incentive for OA journals to accept “lesser-quality” research.^9,10^ Additionally, studies have shown an association between extramural funding and willingness to submit to a journal with a higher article processing fee.^10^ As OA journals continue to gain popularity, identifying gaps in dissemination and author characteristics between traditional journals and their OA counterparts becomes increasingly important. Additionally, given that the reputation of a traditional journal can be used to attract readers to its open access counterpart, researching the dissemination of both publications serves as an opportunity to examine how successful the development of OA journals can be in improving the dissemination of research in a particular field.

To fill this knowledge gap, we aimed to investigate differences in the dissemination of research published in *PRS* and *PRS-GO*. Specifically, we aimed to (1) evaluate differences in the traditional and social media dissemination of research between these two journals, and (2) to identify differences in the author characteristics between *PRS* and *PRS-GO*. We hypothesize that social media will serve as a major driver of dissemination for both publications given the era of information sharing via the internet.

## METHODS

### Database

We extracted data from *PRS* and *PRS-GO* using the Altmetric Explorer database. Altmetric Explorer is a free database available to researchers and provides data on article characteristics, mentions, and AAS. This database was developed to improve transparency on research dissemination.^11^ We extracted articles from both publications using the same 2-year time period (January 1, 2018 to January 1, 2020). We used this period to capture recent articles in the analysis as dissemination of research has likely changed over time. Any publications in the traditional journal, *PRS*, that were OA were excluded to ensure homogeneity. Data were extracted from Altmetric Explorer in April of 2020.

### Primary Outcomes and Categorization of Dissemination

Our main outcomes were AAS, social media mentions, and traditional mentions. AAS is a measure of attention that each publication receives. This score is calculated using social media mentions, mentions of the article in other peer-reviewed manuscripts, news-related press, among other mentions.^12^ News stories included in this score are in the form of online newspapers, magazines, or online stories. We use the word *dissemination* to refer to the number of times a particular research article is mentioned in various platforms. This is used as a proxy for information distribution in this article. Dissemination was categorized as (1) traditional dissemination or (2) social media dissemination to permit us to examine differences in the influence of social media on dissemination for these two different publication models. Traditional dissemination included citations, news, and policy mentions, whereas social media dissemination included Facebook and Twitter mentions.

### Variables

Our research team extracted data regarding basic article characteristics, dissemination statistics, and AAS. Basic article characteristics included article title, author affiliation, author funding, honor roll status of corresponding author, and World Bank country income classification for the country of the corresponding author. We classified honor roll status using the U.S. News Best Hospitals Honor Roll and Medical Specialties Rankings for 2020 and 2021 to identify whether belonging to a ranked institution influenced the dissemination of plastic surgery research.^13^ Additionally, we collected funding as a binary variable, and then we classified funding as (1) National Institutes of Health or (2) plastic surgery organizations (Plastic Surgery Foundation, American Association of Plastic Surgeons, American Society for Surgery of the Hand, among others). We then collected data regarding World Bank country income classification, which included high-income, upper-middle income, lower-middle income, or low-income.^14^ If there were any missing data for author affiliation or funding, two members of the research team manually found the affiliation or funding source by downloading the article. This study received exempt status from the University of Michigan’s institutional review board because it is a publicly available database.

### Statistical Analysis

We used descriptive statistics to identify differences in article characteristics, article dissemination, and AAS using Wilcoxon rank sum and Chi-square tests. Wilcoxon rank sum tests were used for continuous variables given the skewed nature of the data, and Chi-square tests were used for categorical variables.

We then used multivariable linear regression models to examine the association of author and journal characteristics and the three dissemination outcomes (AAS score, social media mentions, and traditional mentions). In each model, we controlled for journal type, time (in months), whether the corresponding author was affiliated with an honor roll hospital, whether the authors declared grant funding for the publication, and World Bank country income classification. We then used the postestimation marginal effects to determine the predicted AAS score, social media mentions, and traditional mentions. Significance was set at a *P* value less than 0.05. All analyses were performed using Stata 15.0 (StataCorp. 2017. College Station, Tex.: StataCorp LLC).

## Results

### Basic Characteristics

Of the total 1798 articles included in the analysis from January 1, 2018 to January 1, 2020, 1031 were from *PRS,* and 767 were from *PRS-GO*. The average AAS per article was higher for *PRS* (15.2) compared with *PRS-GO* (8.1), indicating approximately double the attention and dissemination for *PRS*. In *PRS*, the average number of social media mentions per article was 21.5 (SD ± 34.5), and the average number of traditional mentions was 4.16 (SD ± 6.9). For *PRS-GO*, the average number of social media mentions per article was 17.9 (SD ± 21.21), and the average number of traditional mentions was 1.19 (SD ± 2.5). Additionally, most of the articles from each of *PRS* and *PRS-GO* had corresponding authors from high-income countries. Table 1 includes an overview of basic characteristics and metrics of articles included in the analysis.

**Table 1.** Basic Journal Characteristics and Metrics

	Total (N = 1798)		*PRS* (N = 1031)		*PRS-GO* (N = 767)		*P* *
	Average (SD)		Average (SD)		Average (SD)		
Mean AAS (SD)	12.2	(29.5)	15.2	(37.8)	8.1	(9.4)	0.35
Mean social media mentions (SD)	19.9	(29.6)	21.5	(34.5)	17.9	(21.2)	0.17
Mean traditional mentions (SD)	2.9	(5.7)	4.2	(6.9)	1.2	(2.5)	<0.001
	N	(%)	N	(%)	N	(%)	
Honor roll hospital	282.0	(15.7)	196	(19.01)	86	(11.2)	<0.001
World Bank income classification							0.002
High-income	1,634	(90.9)	945	(91.7)	689	(89.8)	
Upper-middle income	144	(8.0)	83	(8.1)	61	(8.0)	
Lower-middle income	19	(1.1)	3	(0.3)	16	(2.1)	
Low-income	1	(0.1)	0	(0)	1	(0.1)	
Funding†	391	(21.8)	248	(24.1)	143	(18.6)	0.006
NIH	92	(5.1)	73	(7.1)	19	(2.5)	<0001
Foundation	22	(1.2)	12	(1.2)	10	(1.3)	0.37

*Wilcoxon rank sum test used for continuous variables and chi-square for categorical.

†Foundation funding includes internationally recognized plastic surgery organizations. These include Plastic Surgery Foundation, American Society for Surgery of Hand, American Association for Hand Surgery, among others.

### Predictors of Dissemination

#### Altmetric Attention Score

After controlling for author characteristics, articles in *PRS* had significantly higher AAS scores than articles in *PRS-GO* (β-coefficient: 7.50, 95% CI: 4.75, 10.25, *P* < 0.001) with predicted AAS scores for *PRS* of 15.4 (95% CI: 13.6–17.2) compared with 7.9 for *PRS-GO* (95% CI: 5.8–10.0). However, compared with articles with no funding source, articles with funding had on average AAS scores that were three points lower (β-coefficient: −3.36, 95% CI: −6.64, −0.09, *P* = 0.04). Honor roll status of corresponding author institution did not significantly influence the AAS score of an article. Table 2 illustrates the influence of predictor variables on AAS scores.

**Table 2.** Predictors of AAS

	Beta Coefficient (95% CI)		*P*	Predicted AAS (95% CI)*	
Each month†	−0.49	(−0.68, −0.30)	<0.001		
Journal					
*PRS-GO*	1.00	Reference	—	7.90	(5.83, 9.97)
*PRS*	7.50	(4.75, 10.25)	<0.001	15.40	(13.62, 17.18)
Honor roll status					
No	1.00	Reference	—	11.93	(10.47, 13.40)
Yes	1.71	(−2.05, 5.47)	0.37	13.64	(10.20, 17.09)
Funding status‡					
No	1.00	Reference	—	12.93	(11.41, 14.52)
Yes	−3.36	(6.64, −0.09)	0.04	9.57	(6.68, 12.46)
World Bank income classification					
High−income country	1.00	Reference	—	1231.00	(10.90, 13.72)
Upper−middle income country	−1.65	(−6.65, 3.35)	0.52	10.66	(5.87, 15.45)
Low−middle income country	2.24	(−10.97, 15.45)	0.74	14.55	(1.42, 27.68)
Low−income country	−2.94	(−59.94, 54.06)	0.92	9.37	(−47.61, 66.35)

*Predicted AAS was calculated with the margins command to get the average marginal effects. This is the predicted AAS score after controlling for the other variables.

†We included this predictor to identify whether time influences dissemination. Because months are continuous, this would mean that for every later month, the beta-coefficient represents the proportional increase or decrease in score.

‡All funding sources, regardless of if they were funded by NIH, national plastic surgery organizations, or country-specific organizations, were included in this model.

#### Traditional Mentions

The average number of traditional mentions, including scientific citations, was higher for *PRS* compared with *PRS-GO* (Table 1). After controlling for author characteristics, articles in *PRS* had significantly higher traditional mentions compared with those in *PRS-GO* (β-coefficient: 3.11, 95% CI: 2.62, 3.60, *P* < 0.001). Moreover, articles with honor roll status were associated with greater traditional mentions compared with articles without, but this was not a significant result. Table 3 demonstrates the effect of predictor variables on traditional means of dissemination. The World Bank income classification had no significant effect on dissemination.

**Table 3.** Predictors of Traditional Mentions

	Beta Coefficient (95% CI)		*P*
Each month*	−0.24	(−0.27, −0.21)	<0.001
Journal			
*PRS-GO*	1.00	Reference	—
*PRS*	3.11	(2.62, 3.60)	<0.001
Honor roll status			
No	1.00	Reference	—
Yes	0.74	(0.07, 1.41)	0.03
Funding status†			
No	1.00	Reference	—
Yes	−0.42	(−1.00, 0.17)	0.16
World Bank income classification			
High-income country	1.00	Reference	—
Upper-middle income country	−0.20	(−1.10, 0.69)	0.66
Low-middle income country	0.61	(−1.75, 2.97)	0.61
Low-income country	−0.35	(−10.53, 9.83)	0.95

*We included this predictor to identify whether time influences dissemination. Because months are continuous, this would mean that for every later month, the beta-coefficient represents the proportional increase or decrease in score.

†All funding sources, regardless of if they were funded by NIH, national plastic surgery organizations, or country-specific organizations, were included in this model.

#### Social Media Mentions

Social media mentions for articles in *PRS* were greater than social media mentions for articles in *PRS-GO*; however, this result was not significant (Table 4). Additionally, articles with funding had significantly higher social media mentions compared with articles without funding (β-coefficient: −6.60, 95% CI: −9.88, −3.32, *P* < 0.001). Articles with corresponding authors at an honor roll institution had higher social media mentions as well.

**Table 4.** Predictors of Social Media Mentions

	Beta Coefficient (95% CI)		*P*
Each month*	−0.68	(−0.87, −0.49)	<0.001
Journal			
*PRS-GO*	1.00	Reference	—
*PRS*	4.38	(1.63, 7.13)	0.73
Honor roll status			
No	1.00	Reference	—
Yes	2.66	(−1.11, 6.42)	0.17
Funding status†			
No	1.00	Reference	—
Yes	−6.60	(−9.88, −3.32)	<0.001
World Bank income classification			
High-income country	1.00	Reference	—
Upper-middle income country	−0.72	(−5.72, 4.28)	0.52
Low-middle income country	9.39	(−3.83, 22.62)	0.74
Low-income country	−7.27	(−64.35, 49.80)	0.92

*We included this predictor to identify whether time influences dissemination. Because months are continuous, this would mean that for every later month, the beta-coefficient represents the proportional increase or decrease in score.

†All funding sources, regardless of if they were funded by NIH, national plastic surgery organizations, or country-specific organizations, were included in this model.

#### Dissemination over Time

**Fig. 1.**
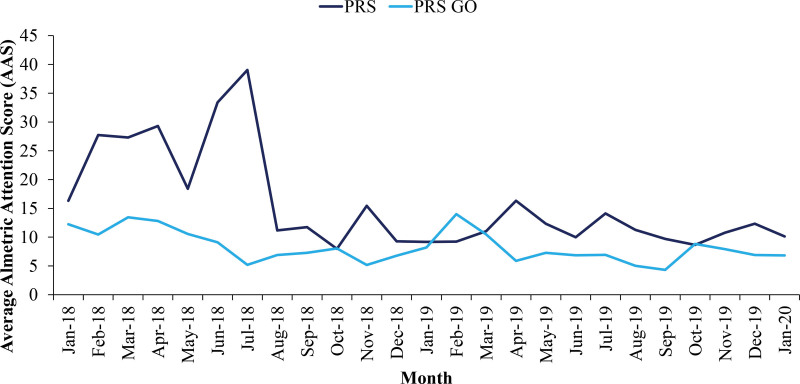
AAS over time.

We found that for every later month an article was published, there was a decrease in AAS score, traditional mentions, and social media mentions. Additionally, the average AAS scores for articles in *PRS* were greater than the AAS scores for *PRS-GO* every month during the time period examined, except February 2019. We hypothesize this may be due to a particular publication or perhaps increased social media outreach in the particular time period. Nonetheless, the magnitude of this difference lessened over time (Fig. 1).

## DISCUSSION

In this analysis of the dissemination of plastic surgery research, we found that dissemination was greater for articles in *PRS* compared with *PRS-GO* and that *PRS* had higher AAS scores from January 1, 2018, to January 1, 2020. Nonetheless, for more recent articles, AAS was comparable for both *PRS* and *PRS-GO*. Additionally, articles with corresponding authors at honor roll institutions had higher measures of traditional dissemination, but not higher AAS scores due to the lack of social media mentions. Our findings highlight the importance of diverse strategies for dissemination, including the recruitment of intentional research that are of interest to the readers. Additionally, this study highlights the role of social media to further promote the dissemination of evidence-based medicine within plastic surgery.

The OA movement was established with the goal of improving dissemination of evidence-based medicine in plastic surgery regardless of pay status.^15^ Furthermore, the COVID-19 pandemic has echoed the importance of well-designed, free, and OA educational materials in medicine.^16,17^ This pandemic has revealed the effect of rapid and reliable information to provide necessary care; thus, OA journals offer the unique opportunity to disseminate new research findings to all providers regardless of subscriptions. Individuals in support of the OA movement argue that to make progress in various scientific disciplines, research needs to be easily accessed.^18^ Specifically, one goal of *PRS-GO* is to disseminate high-quality plastic surgery research to “the widest possible global audience.”^19^ Our findings suggest that although articles from *PRS-GO* have been mentioned in various platforms of dissemination, articles in *PRS-GO* are not being disseminated to the same extent as *PRS*. Moreover, a goal of the OA movement is to help improve scientific communication.^20^ In this study, we found that social media mentions for articles in *PRS-GO* were less than those for *PRS.* Social media is a powerful tool to facilitate communication with scientists in different parts of the world.^21^ Therefore, harnessing the potential of social media to promote articles from OA journals may help foster collaboration and communication among researchers and physicians in different countries, helping meet an additional goal of the OA movement. Additionally, qualitative research to establish high-priority research topics for OA journals may help create content that is of increased interest to readers, helping promote the reputation and attractiveness of a relatively new journal.

Research can be disseminated through social media using nontraditional means. The literature has echoed the importance of social media in disseminating clinical knowledge in medicine and, specifically, in plastic surgery.^22,23^ Recently, visual abstracts have become a way for researchers to creatively and efficiently share their new research findings by creating a visual representation of the research with distilled information similar to what is in a traditional abstract.^24–27^ Hou et al examined if visual abstracts promoted on social media influenced the number of literature citations.^28^ The authors found that although the number of citations were not different from articles with visual abstracts on social media, they did have higher AAS and increased readership. Furthermore, online journal clubs can be used to facilitate dialogue and attract attention to specific articles and publications as a whole. *PRS* selects three articles each month for a journal club, each of which are paired with podcasts on numerous platforms.^29^ Traditional and OA journals can adopt a similar framework to further disseminate publications and expand journal clubs to include social media platforms. For instance, hosting journal clubs through the Instagram Live feature may facilitate further discussion on particular publications. Subsequent analyses may examine the journal club participants and country of origin to shed light on the effectiveness of such initiatives used to promote more global plastic surgery dissemination. Researchers should utilize visual abstracts, in addition to other creative methods, to communicate their research through social media. Individual journals could create roles for designated officials to promote publications through social media via visual abstracts, twitter mentions, and journal clubs. Future research may focus on identifying the extent to which individuals accessing articles posted on social media platforms consume the research. Moreover, social media may serve as an avenue to disseminate knowledge beyond providers and directly to patients. Sedrak et al conducted a content analysis of Twitter search engine results for the terms “lung cancer” and found that although some tweets discussed clinical trials, none of them provided links or instructions for enrollment.^30^ The authors suggest that social media can be used as a medium to connect researchers with patients. A similar framework can be used to connect physicians with patients, disseminate knowledge on postoperative management, and recruit participants for plastic surgery research studies.

Numerous factors contribute to the dissemination of plastic surgery research. The reputation of a particular journal will drive readership to the articles published in any given issue, helping disseminate the research. Furthermore, article characteristics contribute to the popularity of a particular article. These characteristics may include the article’s abstract, title, graphics, tables, figures, and videos. For example, shorter titles featuring keywords are probably more likely to be discovered.^31,32^ Relevant and high-quality graphics and organization may drive additional readership to a particular publication. Therefore, differences in dissemination of research may be partially attributable to the specific resources a publication allocates to an article. Future research may shed light on whether reported differences in dissemination could also be explained by differences in the investment in promotion.

OA publications have the potential to advance the field of science by facilitating further research and scientific collaboration. Despite the potential of OA publications to facilitate collaboration, the OA model provides an opportunity for certain publications to abuse the framework and make money off authors. Predatory journals may promise a peer-review process but provide low-quality or no peer reviews for submitted articles. Additionally, articles from a subset of OA journals may not disclose whether the journals are indexed in databases such as PubMed or the Directory of Open Access Journals.^33^ The index status of a journal can contribute to the credibility and the dissemination of scientific literature. For example, *PRS-GO* is indexed on PubMed Central, Directory of Open Access Journals, Scopus, Emerging Sources Citation Index, and Google Scholar.^19^ Additionally, *PRS-GO* offers a thorough scientific review to elevate the quality of work accepted and provide the readers with valuable literature. The dissemination of OA articles through less scientific modalities, for example a social media outlet, can be dangerous in the era of misinformation. Nonetheless, social media has provided researchers an opportunity to disseminate their work and discuss topics in medicine with other healthcare professionals in a way that would have otherwise not been possible. Journals and medical societies should provide guidelines on how to properly assess a social media post for misinformation to help educate healthcare professionals on effectively using social media for academic purposes. Future initiatives may focus on developing an interface to permit the rapid exchange of medical knowledge through a social media platform, with features for fact checking, and quick links to recent and reliable scientific publications. Additionally, outreach may focus on educating authors and readers on the benefits of OA journals, which are commonly viewed as research of lesser quality.

Gathering research from countries in lower-income settings is vital to foster the development of a robust understanding of plastic surgical conditions worldwide. The Global Forum for Health Research has emphasized the gap in research between high-income countries and low-middle-income countries.^34^ Researchers have echoed the lack of contribution from low- and middle-income countries to research in various fields.^35–37^ For example, in an analysis of research published in leading general psychiatry journals, the authors found that less than 4% of the research published in leading psychiatry journals was from low- and middle-income countries.^35^ Our study found a similar phenomenon: in two of the most prominent plastic surgery publications, the proportion of publications coming from low- and middle-income countries was significantly lower than the proportion of publications from high-income and upper-middle income countries. Encouraging healthcare administrations in low- and middle-income countries to develop research capacity in their facilities is needed to increase the number of publications coming from low- and middle-income countries. Strategies may include developing online modules and research symposiums focused on plastic surgery research building in low-resource settings. Furthermore, this study showed that authors from institutions perceived as being more prestigious had more dissemination of their published research. Therefore, certain initiatives focused on providing certain researchers (institutions with less resources who produce high-quality, cutting-edge research) a reduced publishing fee for submission to an OA journal may promote a more equitable publishing environment. A more diverse array of publications in the plastic surgery research will provide global health policy makers more data to better develop strategies to help address the disproportionate global burden of disease. Additionally, the diverse perspective of plastic surgeons in lower resource countries may provide physicians in higher income countries with unique techniques and approaches to the management of various plastic surgical conditions.

Our study has limitations. We recognize the data provided in the Altmetric Explorer database change as additional data become available; thus, our results reflect the metrics on the day it was extracted from the database. Nonetheless, we included a 2-year time frame to ensure that both prior and recent articles were included in the analysis. Future research on longitudinal dissemination of publications in plastic surgery may shed light on the trends in dissemination among journals. In the Altmetric Explorer database, the news mentions include only online sources; however, this is the main modality of information consumption in the current era. Additionally, dissemination is a broad term that can be defined in numerous ways; however, we measured dissemination using three different metrics (AAS, traditional mentions, social media mentions) to develop a comprehensive understanding of plastic surgery research dissemination in *PRS* and *PRS-GO*. We did not include all social media platforms in our analysis of social media dissemination because data from some social media platforms, like Instagram, were added to the database after the start of our study period. Additionally, other international social media platforms (including WhatsApp and Vkontakte) were not included as variables in the database and could not be analyzed. Nonetheless, Twitter and Facebook have been described as social media outlets used in plastic surgery to facilitate dialogue about the field.^38^ Altmetric does list categories for each article; however, too many of this variable were missing to include it in our final analysis. Additionally, this is a cross-sectional observational study; so we cannot claim causation. This study is subject to the inherent biases of observational data. In our categorization of funding, we classified funding in two ways: (1) as a binary variable to include any type of funding mentioned in the acknowledgements of the article and (2) as funding related to prestigious funding sources in the field of plastic surgery. Prestigious funding sources were limited to National Institutes of Health and globally recognized plastic surgery organizations and not stratified by income classification for the country. Furthermore, the quality of the articles included in our analysis was not assessed. Forthcoming research may focus on identifying how the quality of articles published in *PRS* and *PRS-GO* contribute to AAS and dissemination. Altmetric does not provide a variable depicting the amount of time each individual spends on a particular website or article. Additional research may examine the type of readers consuming the articles on social media platforms to ensure the appropriate audience is reached. Moreover, Altmetric does not contain data regarding articles that were originally submitted to *PRS* and then subsequently went to *PRS-GO*. Subsequent analyses may shed light on the characteristics of articles submitted to *PRS* and *PRS-GO*.

## CONCLUSIONS

As the methods for consuming medical information continue to evolve, it becomes increasingly important to utilize the various strategies that maximize scientific dissemination. Despite these limitations, this study reveals the importance of social media in the dissemination of plastic surgery research, regardless of affiliated institution or funding status. Additionally, policies aimed at promoting publications from certain authors in low- and middle-income countries or authors belonging to institutions with less resources who produce high-quality research may increase the diversity of research in plastic surgery literature. Future research may investigate the information-seeking behavior of plastic surgery healthcare professionals, at all levels of training, to identify potential strategies to promote the effective and efficient consumption research.

## ACKNOWLEDGMENT

The authors thank Altmetric for supplying the data for this study. They also thank Melissa Beyrand for her help with data extraction

## References

[1] Budapest Open Access Initiative. Read the Declaration. Available at https://www.budapestopenaccessinitiative.org/read. Published 2022. Accessed February 18, 2022.

[2] Frankland J Ray MA. Traditional versus open access scholarly journal publishing: an economic perspective. J Sch Publishing. 2017;49:5–25.

[3] Björk BC Solomon D. Open access versus subscription journals: a comparison of scientific impact. BMC Med. 2012;10:73. 22805105 10.1186/1741-7015-10-73 PMC3398850

[4] Boyd CJ Ananthasekar S Kurapati S. Examining the correlation between altmetric score and citations in the plastic surgery literature. Plast Reconstr Surg. 2020;146:808e–815e. 33234981 10.1097/PRS.0000000000007378

[5] Asaad M Howell SM Rajesh A. Altmetrics in plastic surgery journals: does it correlate with citation count? Aesthet Surg J. 2020;40:NP628–NP635. 32506129 10.1093/asj/sjaa158

[6] American Society of Plastic Surgeons. Plastic and Reconstructive Surgery Journal. 2020. Available at https://dergipark.org.tr/en/pub/tid/page/5833. Accessed June 1, 2020.

[7] Rohrich RJ Weinstein AG Moore ID. PRS global open: a dream turning to reality. Plast Reconstr Surg Glob Open. 2020;8:e3395. 33425633 10.1097/GOX.0000000000003395 PMC7787301

[8] Quinlan CS Collins AM Nason GJ. The use of social media by plastic surgery journals. J Plast Reconstr Aesthet Surg. 2016;69:1009–1011. 27210579 10.1016/j.bjps.2016.04.004

[9] Freshwater MF. Open access, fauxpen access: problems in transparency and proposed solutions. J Plast Reconstr Aesthet Surg. 2014;67:589–590. 24630568 10.1016/j.bjps.2013.09.035

[10] Solomon DJ Björk BC. Publication fees in open access publishing: Sources of funding and factors influencing choice of journal. J Am Soc Inf Sci Technol. 2012;63:98–107.

[11] Altmetric. Insights across the entire Altmetric database. Available at https://www.altmetric.com/products/explorer-for-publishers. Accessed November 1, 2021.

[12] Elmore SA. The Altmetric Attention Score: What does it Mean And Why Should I care? Los Angeles, Calif.: SAGE Publications; 2018. 10.1177/0192623318758294 PMC5912977 29448902

[13] US News. U.S. News Best Hospitals Honor Roll and Medical Specialties Rankings for 2020 to 2021. Available at https://health.usnews.com/health-care/best-hospitals/articles/besthospitals-honor-roll-and-overview. Published 2020. Accessed November 1, 2021.

[14] World Bank. World Bank country and lending groups. Available at https://datahelpdesk.worldbank.org/knowledgebase/articles/906519-world-bank-country-and-lending-groups. Published 2020. Accessed December 1, 2020.

[15] Laakso M Welling P Bukvova H. The development of open access journal publishing from 1993 to 2009. PLoS One. 2011;6:e20961. 21695139 10.1371/journal.pone.0020961 PMC3113847

[16] Chan AKM Nickson CP Rudolph JW. Social media for rapid knowledge dissemination: early experience from the COVID-19 pandemic. Anaesthesia. 2020;75:1579–1582. 32227594 10.1111/anae.15057 PMC7228334

[17] Vervoort D Ma X Luc JGY. Rapid scholarly dissemination and cardiovascular community engagement to combat the infodemic of the COVID-19 Pandemic. Can J Cardiol. 2020;36:969.e1–969.e2. 10.1016/j.cjca.2020.03.042 PMC7270554 32299782

[18] Quinn MM. Open access in scholarly publishing: Embracing principles and avoiding pitfalls. Ser Libr 2015;69:58–69.

[19] Plastic and Reconstructive Surgery – Global Open. About Plastic and Reconstructive Surgery – Global Open. 2020. https://journals.lww.com/prsgo/Pages/aboutthejournal.aspx. Accessed October 1, 2020.

[20] Pöschl U Koop T. Interactive open access publishing and collaborative peer review for improved scientific communication and quality assurance. Inf Serv Use. 2008;28:105–107.

[21] Sun H Puterbaugh MD. Using social media to promote international collaboration. Pennsylvania Libraries. 2013;1:60–74.

[22] Johannsson H Selak T. Dissemination of medical publications on social media–is it the new standard? Anaesthesia. 2020;75:155–157. 31338831 10.1111/anae.14780

[23] Zhou JZ Lemelman BT Done N. Social Media and the dissemination of research: insights from the most widely circulated articles in plastic surgery. Plast Reconstr Surg. 2018;142:555–561. 30045187 10.1097/PRS.0000000000004598

[24] Nikolian VC Ibrahim AM. What does the future hold for scientific journals? visual abstracts and other tools for communicating research. Clin Colon Rectal Surg. 2017;30:252–258. 28924398 10.1055/s-0037-1604253 PMC5595539

[25] Gloviczki P Lawrence PF. Visual abstracts bring key message of scientific research. J Vasc Surg. 2018;67:1319–1320. 29685244 10.1016/j.jvs.2018.04.003

[26] Ibrahim AM Lillemoe KD Klingensmith ME. Visual abstracts to disseminate research on social media: a prospective, case-control crossover Study. Ann Surg. 2017;266:e46–e48. 28448382 10.1097/SLA.0000000000002277

[27] Ramos E Concepcion BP. Visual abstracts: redesigning the landscape of research dissemination. Semin Nephrol. 2020;40:291–297. 32560778 10.1016/j.semnephrol.2020.04.008

[28] Hou LT Pierorazio PM Koo K. Impact of Social Media Visual Abstracts on Media Attention and Literature Citation in Urology. Philadelphia, Pa.: Wolters Kluwer; 2020. 10.1097/JU.0000000000001136 32516017

[29] Plastic and Reconstructive Surgery Journal. PRS Journal Club. 2020. Available at https://journals.lww.com/plasreconsurg/pages/podcastepisodes.aspx?podcastid=5. Accessed November 15, 2020.

[30] Sedrak MS Cohen RB Merchant RM. Cancer communication in the social media age. JAMA Oncol. 2016;2:822–823. 26940041 10.1001/jamaoncol.2015.5475

[31] Deng B. Papers with shorter titles get more citations. Nature. 2015.

[32] Letchford A Moat HS Preis T. The advantage of short paper titles. R Soc Open Sci. 2015;2:150266. 26361556 10.1098/rsos.150266 PMC4555861

[33] Memon AR. Revisiting the term predatory open access publishing. J Korean Med Sci. 2019;34:e99. 30950249 10.3346/jkms.2019.34.e99 PMC6449603

[34] Davey S. The 10/90 Report on Health Research 2003–2004. Geneva, Switzerland: Global Forum for Health Research; 2004.

[35] Patel V Kim YR. Contribution of low-and middle-income countries to research published in leading general psychiatry journals, 2002–2004. Br J Psychiatry. 2007;190:77–78. 17197661 10.1192/bjp.bp.106.025692

[36] Pastrana T Vallath N Mastrojohn J. Disparities in the contribution of low- and middle-income countries to palliative care research. J Pain Symptom Manage. 2010;39:54–68. 19892510 10.1016/j.jpainsymman.2009.05.023

[37] Sumathipala A Siribaddana S Patel V. Under-representation of developing countries in the research literature: ethical issues arising from a survey of five leading medical journals. BMC Med Ethics. 2004;5:E5. 15461820 10.1186/1472-6939-5-5 PMC524359

[38] Gould DJ Leland HA Ho AL. Emerging trends in social media and plastic surgery. Ann Transl Med. 2016;4:455. 28090511 10.21037/atm.2016.12.17 PMC5220023

